# A public panel reviews applications and questions applicants: Team member and public contributor discuss a transparent and inclusive approach to data access reviews

**DOI:** 10.23889/ijpds.v8i2.2249

**Published:** 2023-09-14

**Authors:** Kirsteen Campbell, Rebecca Whitehorn, Simon Browning, Rebecca Harmston, Ray Harris, Szu-Chia Huang, Dianna Moylan, Karen Williams, Jacqueline Oakley, Katharine Evans, Stela McLachlan, Richard Thomas, Emma Turner, Robin Flaig, Andy Boyd

**Affiliations:** 1 University of Edinburgh, Edinburgh, United Kingdom; 2 University of Bristol, Bristol, United Kingdom

## Objectives

We created a panel with members of the public and longitudinal study participants who review our data access requests. This panel forms an integral part of our data access application process, giving the public a say who can access the data for research.

## Method

We advertised our lay member vacancies using social media, newsletters, word of mouth and the internet. We appointed six people to the public panel. Our panel includes study participants, NHS service users, parents, carers, and people with experience of disability, neurodiversity, and long-term health conditions.

The Panel Terms of Reference were created with help from stakeholders and study teams involved in longitudinal studies that involve the public in data access applications. This ensured that the purpose of the panel was clear. The panel reviews lay summaries and makes sure that researchers have adequate public involvement in their project.

## Results

Panel members have reviewed 28 applications. Researchers present their research at an online meeting with the panel then answer questions from the panel members. We publish meeting minutes on our website for transparency.

A 6-month review was overwhelmingly positive - all panel members indicated they felt valued. They felt able to challenge and question researchers as part of the data access application process. This provides a level of public scrutiny to our work.

“I feel there’s a real value in the panel. You get a real sense that this has got such potential to make a contribution.” (panel member)

We are further developing the Panel Terms of Reference with panel members. We will consider additional areas of responsibility, for example, public benefit review.

## Conclusion

We regularly review how to improve public involvement in our work. The panel has proven its value during our application process. Therefore we are exploring with the panel a new approach to assess the public benefit of applications and what is meant by ‘public benefit research’.

